# Study of event‐related potentials by withdrawal friction on the fingertip

**DOI:** 10.1111/srt.13232

**Published:** 2022-11-25

**Authors:** Chen Si, Huang Qin, Yang Chuanzhuang, Tang Wei, Xu Lin

**Affiliations:** ^1^ Research Center of Fluid Machinery Engineering and Technology Jiangsu University Zhenjiang China; ^2^ Department of Mechanical Engineering China University of Mining and Technology Xuzhou China

**Keywords:** EEG, event‐related potentials, skin friction, tactile perception

## Abstract

**Objectives:**

The lack of understanding about the brain's reaction processes in perceiving touch and separation between skin and object surfaces is a barrier to the development of existing brain‐computer interface technologies and virtual haptics. These technologies are limited in their ability to advance. It leaves prosthesis users with a limited amount of tactile information that they can feel. This study aims to determine whether distinct surface aspects of various items trigger different reactions from the brain when friction is removed from the surface.

**Methods:**

When friction is suddenly removed from the surface of an item, a technique called event‐related potential, (ERP) is used to study the features of people's EEGs. It is done after the subject has actively explored the object's surface. A 64‐channels EEG collecting system was utilized to acquire EEG data from the individuals. [Corrections added on 5 December 2022, after first online publication: The preceding sentence has been updated.] The event‐related potentials for friction removal were generated using the Oddball paradigm, and the samples consisted of sandpaper with three distinct degrees of roughness. We utilized a total of 20 participants, 10 of whom were male, and 10 of whom were female, with a mean age of 21 years.

**Results:**

It was discovered that the P3 component of event‐related potentials, which is essential for cognition, was noticeably absent in the friction withdrawal response for various roughnesses. It was the case regardless of whether the surface was smooth or rough. Moreover, there was no statistically significant difference between the P1 andP2 components, which suggests that the brain could not recognize the surface properties of objects with varying roughness as the friction withdrawal was being performed.

**Conclusions:**

It has been demonstrated that tactile recognition does not occur after friction withdrawal. The findings of this paper could have significant repercussions for future research involving the study of haptic perception and brain‐computer interaction in prosthetic hands. It is a step toward future research on the mechanisms underlying human tactile perception, so think of it as preparation.

## 1 INTRODUCTION

Touch is an important way for humans to interact with the environment. The tip of the finger skin has no less than 600 nerve fibers per square millimeter, making the finger very sensitive to sensory objects.^1^ When the fingertip skin contacts and rubs against the surface of an object, the interaction of forces will make the skin deform and vibrate. Then, the tactile receptors in the skin encode the stimulation information to generate action potential.^2^ The action potential reaches the somatosensory center of the brain through the neural circuit.^3^ The conduction direction, time, amplitude, and other action potential parameters can reflect the texture information of the object surface.^4^, ^5^

Losing function in one or both hands can result in significant physical infirmity and even mental disability. Each year, thousands of people suffer the consequences of upper‐limb paralysis or of amputation caused by a traumatic event or disease.^6^ Robotic limbs can restore independence to these individuals.^6^ However, the existing technology for the brain‐computer interface can only send the pressure signal to the brain via electrical stimulation whenever the robotic limb makes touch with the outside world. Because the physiological signal that represents the end of touch cannot be sent to the brain, users of robot limbs are unable to feel when they are in contact with external things or when they are no longer in contact with those items. Additionally, present virtual haptic technologies often replicate touch by delivering force stimulation to the user via haptic gloves. This is done in order to provide a more lifelike experience. A perfect virtual haptic technology, on the other hand, would be able to create pertinent reactions by directly activating the brain. It generates the impression of touch and separation between the virtual hand and the item, but it does so without requiring any actual interaction between the hand and the outside world. For the purpose of developing brain computer interface technology and virtual touch technology, which are both expected to restore the sensory information of amputees, it is highly vital to examine the brain reaction generated by the existence and removal of friction.

When does the perception happen? Some people think it happens when a finger is pressed on the object. While others might think it happens after touch.^7^ Delicate tactile sensation depends on the stress and strain of the skin under friction. The question is to discuss whether the surface characteristics of different objects will trigger different brain responses when the friction is withdrawn.

Traditional neuromolecular biology and neurobiochemistry are not enough to observe the brain's response when the sense of touch occurs. The reason is that we cannot think of a change in people's thinking simply as a change in a particular part of the brain tissue, cells, or neurotransmitters. In 1965, Sutton proposed the concept of event‐related potentials (ERPs), recording brain‐evoked potentials on the surface of the skull using average superposition technology to reflect the neurophysiological changes of the brain during the cognitive process.^8^ Because ERPs are closely related to cognitive processes, they are believed to be a pivotal way to explore the brain's secrets.^9^ Since then, ERPs have been widely used in psychophysiology.^10^, ^11^ It is a kind of electroencephalograph (EEG) technology developed based on an EEG.^12^ It has a high time resolution and can detect changes in milliseconds.^9^ Because most of the EEG signal consists of noise, the EEG induced by the object's surface features is quickly submerged in the spontaneous EEG of the brain. The ERP technology can be used to filter out EEG noise very well and obtain EEG signals induced by the frictional resistance of the object's surface.^9^

The direct research on the relationship between surface characteristics and electroencephalogram is still in its infancy. Through a series of experiments, Chen et al.^13^, ^14^, ^15^ found that samples with different surface characteristics have different evoked potentials in the brain. However, when tactile cognition is performed on multiple sets of samples, the amplitude and latency of the same sample's evoked potential are not always consistent with their surface characteristics but are more directly related to the difficulty of the cognitive process. Tang et al.^16^ also used ERP technology to study the width and spacing of texture gratings as well as the amplitude and latency of evoked brain ERPs. They found that the P300 latency is associated with the difference between grating target and smooth nontarget stimuli. The greater the sense of difference, the shorter the latency, the larger the texture grating width, the smaller the spacing, and the larger the P300 peak. However, we have not seen any research on the tactile response of the brain after the stimulus is removed. Therefore, this paper adopts ERP technology to observe the EEG characteristics when friction is withdrawn suddenly after people actively explore the object's surface.

## 2 MATERIALS AND METHODS

### 2.1 Experimental materials and preparation

The experimental samples A, B, and C are three kinds of sandpaper with different roughness: 600 mesh; 1000 mesh; 3000 mesh. The sample is cut to a size of 30 mm × 30 mm.

### 2.2 Experimental methods

**FIGURE 1:**
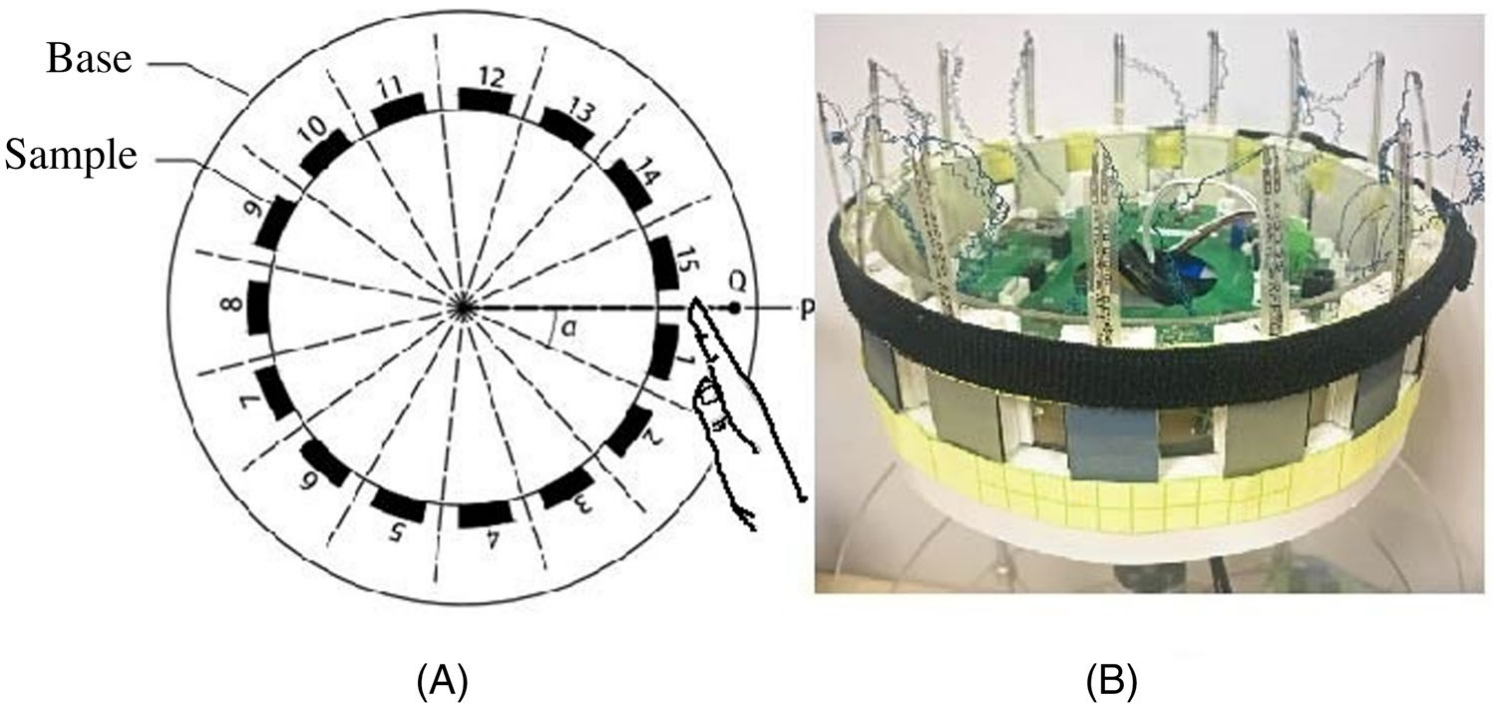
Tactile stimulus presented device. (A) Diagrammatic sketch; (B) Physical picture

The sample is placed in a self‐made tactile stimulation presentation device (Patent Authorization No. CN 108664903 A), as shown in Figure 1 described by reference.^28^ The signal acquisition module consists of 15 soft pressure sensors, an accelerometer, and a torque sensor to collect active pressure, friction, and vibration signals.^28^ The communication transmits these three signals and uses the shaft encoder on the roller to perform position marking.^28^

When each sample is transposed to the specified starting point, a trigger signal is sent to the Electrical Geodesics Incorporated (EGI) 64‐channel ERP acquisition system (EGI; Eugene, OR, USA) via synchronizer (DB9 Pinout).^28^ Each sample is labeled with EEG signals. A sample substrate is made of silicone oil paper covering the pressure sensor all around the roller without interval.^28^ The accelerometer is placed on the right index fingernail cover to measure the vibration signal between the finger and the sample.^28^

The subjects are 21‐year‐old undergraduates or postgraduates, 10 males and 10 females, all right‐handed, and have no hand injury.^28^ Before the experiment started, the subject combed his hair and stayed in the experimental environment for 10 min to move freely. At the beginning of the experiment, subjects were asked to cover their eyes, calm and relax their hands, and place their index fingers close to the sample stimulation presentation device. The subject's task is to perceive the surface roughness of the sample. Because the subjects' arousal status and attention would influence the experiment results, the volunteers were asked to concentrate on the experimental task. The sample replacement sequence is a preprogrammed sample sequence, which is random to the subject. Each experiment required approximately 100 tactile samples for a total of 4–5 min. After resting for 5 min, repeat group 2.

**FIGURE 2:**
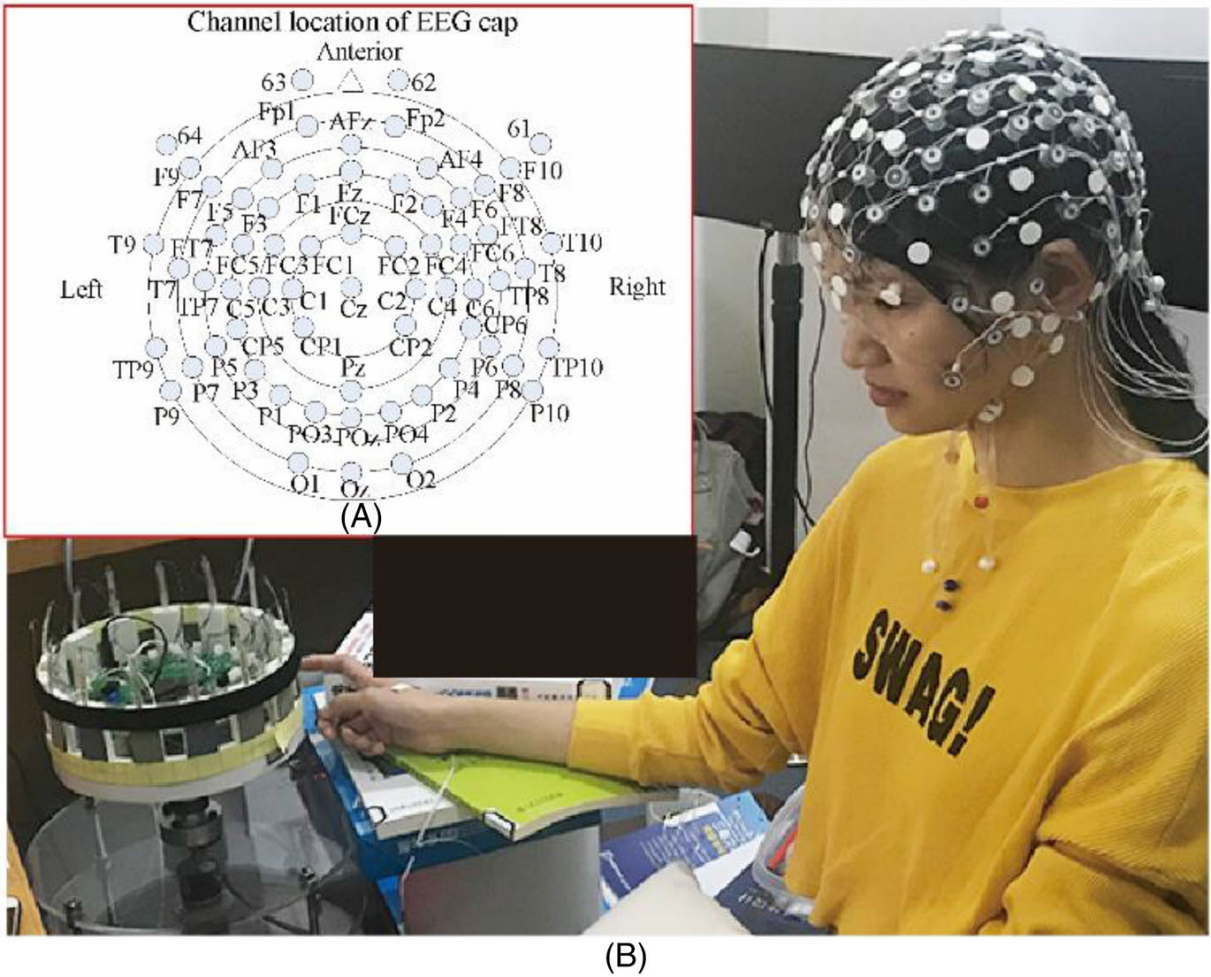
(A) Electrodes' distribution. (B) Experiment scene diagram for the tactile perception of multimodal measurement. [Corrections added on 5 December 2022, after first online publication: Figure 2 legend has been updated.]

**FIGURE 3:**
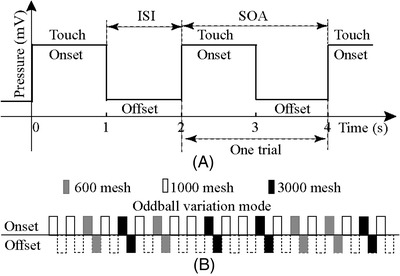
(A) Stimulus^13^ and (B) Oddball variation mode with a stimulus of friction‐onset and ‐offset

The classical experimental mode in ERP experiments include Go/Nogo,^17^ Flanker,^18^ Stroop,^19^ and Oddball.^20^ The Oddball mode^20^ is the most commonly used in psychophysical experiments related to touch, as shown in Figure 2. The Oddball paradigm is the two stimuli that randomly present the same sensory channel in the experiment. The probabilities of the two stimuli differ significantly. High probability is the standard stimulus (non‐target stimulus), and the low probability is deviation stimulus (target stimulus), so that standard stimulus constitutes the background of deviation stimulus. Oddball paradigm can derive many subtypes and produce P300, MMN, and other components related to the difference in stimulus probability, which is widely used. The subject's task is to identify the roughness of the sample surface. The volunteers maintained a constant rhythm for every trial to affect interstimulus interval (ISI) during the experiment, as shown in Figure 3A. After one touch is over, lift finger and start the next touch. The ISI is about 1 s, while stimulus onset asynchrony is about 2 s.^13^ An experimental assistant quietly placed the samples under the volunteers' fingers to avoid disturbing them. [Corrections added on 5 December 2022, after first online publication: The original 3rd paragraph was deleted from 2.2 Experimental Methods.]

### 2.3 Data processing and analysis methods

The experimental data are analyzed offline. The steps are as follows: merge behavior data and EEG preview; removal of electrooculogram artifacts; subparagraph; baseline correction; removal of artifacts; superposition averaging.

In data processing, a bandwidth of 0.05–30 Hz was selected to remove the electrooculogram artifacts. EEG recorded the whole experimental process. The main components of ERPs were analyzed in this paper, including P1, P2 and P3. P1 is a significant positive component with an incubation period of about 100 ms. It usually appears on the occipital side of the temporo‐occipital area and is affected by attention, reflecting the characteristics of physical stimulation. P2 is a significant positive component with a latency of about 200 ms, which reflects task‐related processing. P3 has been studied most for its correlation with cognition. Its families include P3a and P3b, and the incubation period is about 300 ms or even longer.^21^ Among them, P3a can be induced by rare, task‐independent stimuli distributed forward on the scalp. Rare task‐related stimuli can induce P3b, usually distributed in the central parietal region.

## 3 RESULTS AND DISCUSSION

### 3.1 ERP topographic maps

**FIGURE 4:**
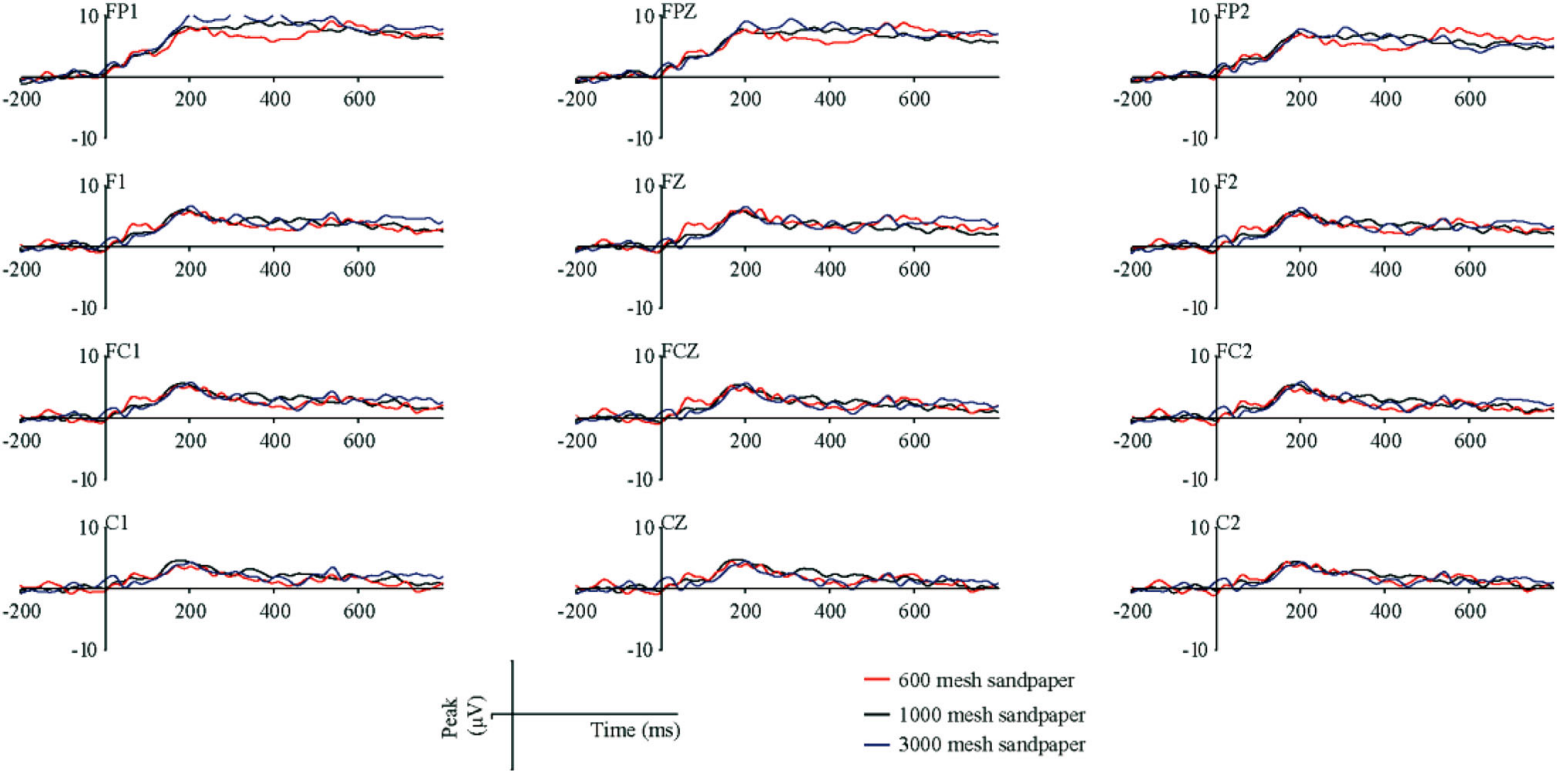
Event‐related potential (ERP) maps of the brain after friction withdrawal from 0 to 600 ms

In the ERP topography shown in Figure 4, positive ERP‐evoked potentials were observed at 100 ms. Large amplitude ERP positive evoked potentials appeared in the frontal, temporal, and parietal lobes at 200 ms. From 300 ms to 600 ms, the brain area that triggers positive ERP evoked potentials gradually narrows to the frontal and temporal lobes. The ERP positive evoked potential induced by 600 mesh sandpaper appeared earlier, with a larger amplitude and longer duration.

### 3.2 ERP characteristics

**FIGURE 5:**
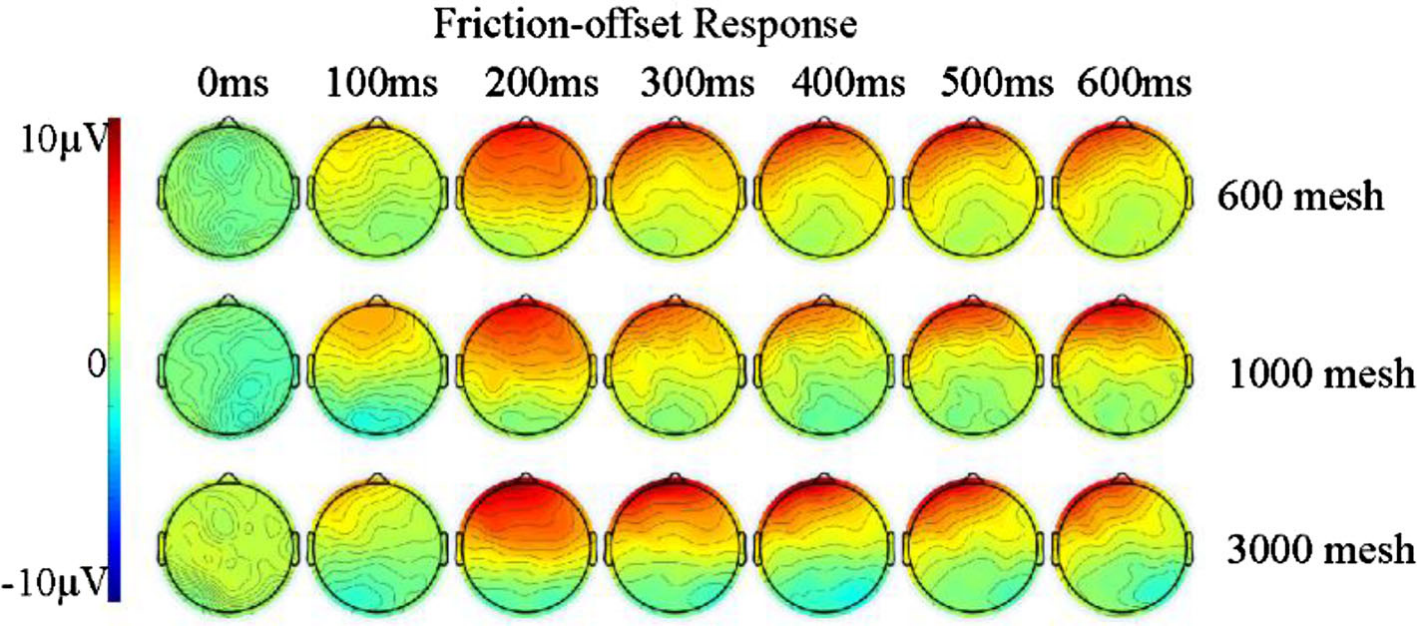
The event‐related potential (ERP) waveforms of the frontal lobe of the brain after friction withdrawal

According to the ERP topographic map, it can be seen that the ERPs induced by the removal of friction are concentrated in the frontal, parietal, and temporal lobes. So, the electrode from the frontal lobe to the parietal lobe was selected, and the typical ERP waveform was observed, as shown in Figure 5. Wave peaks appear at 100 and 200 ms. The ERP of the frontal lobe tends to maintain after 200 ms, but the ERP that gradually transmits to the parietal lobe gradually decreases after 200 ms, making the peak more obvious at 200 ms. But compared with previous research, the difference lies in that the ERPs induced by the withdrawal of friction have no obvious P3 component. The P3 component is related to cognitive activities such as attention, identification, decision‐making, and memory.^22^, ^23^ P3 can respond to the target stimulus, and its amplitude and latency are positively correlated with experimental settings and task difficulty. There is no P3 component in friction withdrawal, which means that the mental process of cognition does not occur.

In practical experience, people often think that cognition occurs after the fingers leave the object's surface. It is more likely due to the cognitive process through memory rather than the cognitive process directly induced by the surface features of objects. Hopfingera et al.^24^ found that the presence and absence of stimuli can cause the brain to strengthen the processing of stimuli, that is, to produce P1 and P2 components. Compared with tactile cognition, when the friction force is removed, the change in friction force disappearing produces P1 and P2 components. However, after the stimulation disappears, there is no attention in the brain, and no cognitive process.

It should be noted that the previous tactile ERP experiments provide evidence that both nontarget and target stimuli could produce P3 components.^14^ However, Hopfinger^24^ found that nontarget visual stimulation did not produce a P3 component. The possible reason is that, unlike vision, the human brain has a relatively short memory for touch. Miller et al. found that short‐term memory capacity is (7 ± 2) units.^25^ This conclusion is mainly based on the study of people's visual and auditory memory characteristics. Based on their research, Liu et al. conducted experiments on tactile memory capacity. They found that people's short‐term tactile memory capacity was no more than 7, with an average of 3 or 4.^26^ This shows that the capacity of tactile memory is smaller than that of visual and auditory memory, and the retention time of tactile memory is shorter. We speculate that because the brain has a hard time remembering tactile target stimulation and needs to re‐recognize it every time the stimulation comes, the P3 component can also be produced for non‐target stimulation.

**FIGURE 6:**
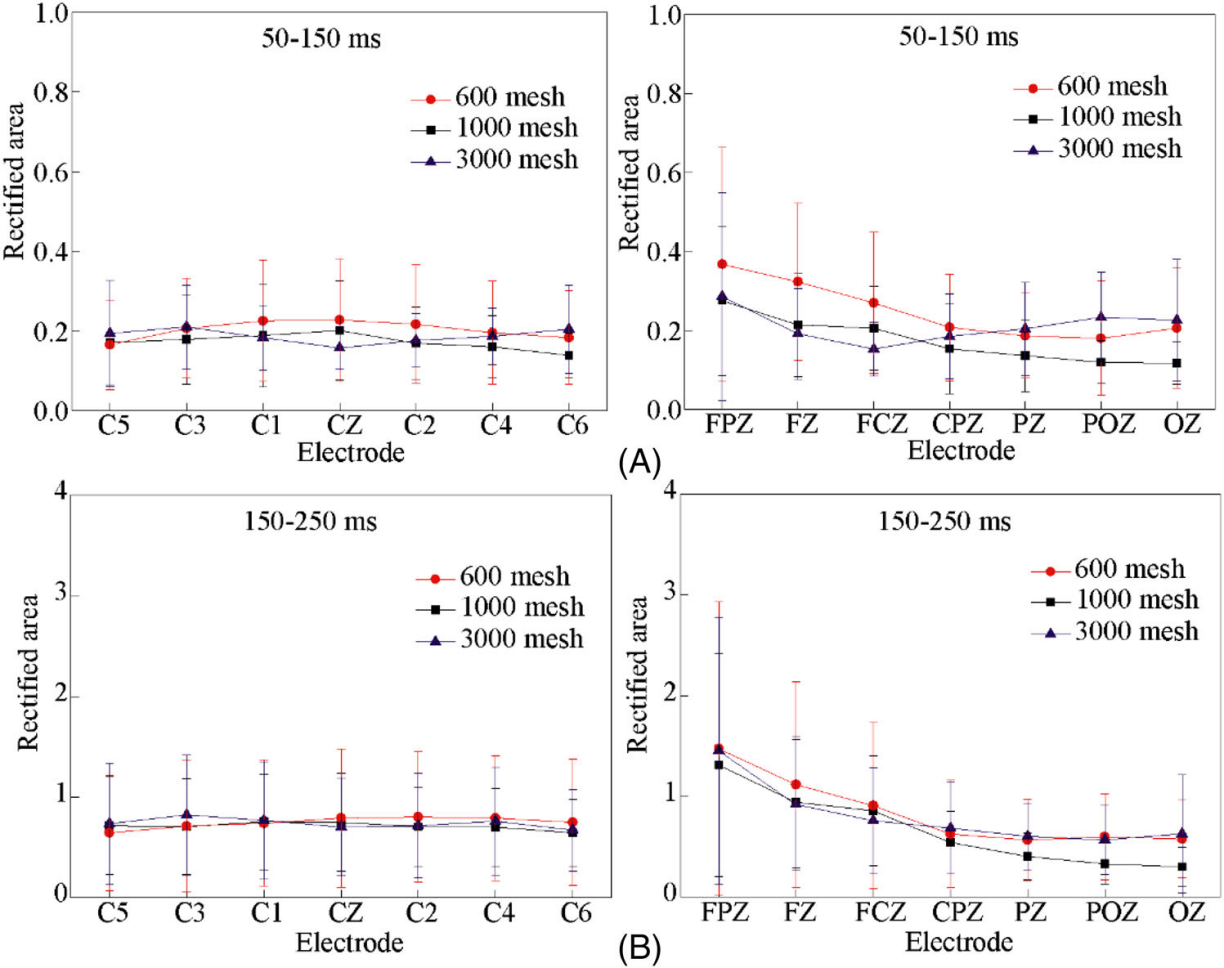
The rectified area of P1 and P2 after friction withdrawal

**FIGURE 7:**
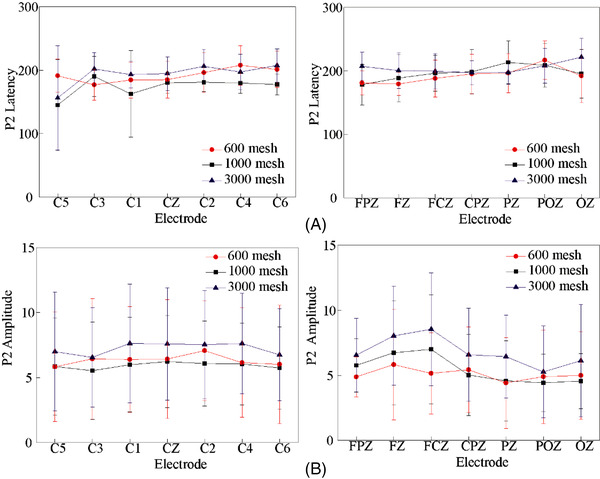
The amplitude and latency of P2 after friction withdrawal

Figure 6 shows the rectified area at 50 ms before and after P1 and P2. We selected a total of 14 electrodes on the coronal and sagittal lines that are representative of the response of the entire brain for observation, including C1, C2, C3, C4, C5, C6, CZ, FPZ, FCZ, FZ, CPZ, PZ, POZ, OZ. It can be found that there is no significant difference between the rectified area of P1 and P2 components induced by these three samples. However, the rectified areas of P1 and P2 components induced by 600 mesh sandpaper were slightly larger than those induced by 1000 and 3000 mesh sandpaper. Note that 600 mesh has the highest roughness and more obvious stimulation, which may cause its physical characteristics to generate greater irritation. After the friction is removed, the skin pressure and strain are significantly different, resulting in a more obvious response in the brain. But this is just a brain response caused by physical stimulation, not a cognitive process. However, in some literature, P2 is also thought to be associated with early cognition.^27^ So we analyze the amplitude and latency of the P2 component, as shown in Figure 7. The amplitude of the P2 component induced by the friction withdrawal of the three sandpaper samples is almost the same, and there is no significant difference. Although the average value of the P2 peak caused by 3000 mesh sandpapers is slightly higher than by the other two samples, there is no statistical difference.

Current brain‐computer interface technology cannot make the brain recognize the feeling of separation between the prosthetic limb and the object surface. If we want to express the surface features of virtual objects and use bionic skin to “read” the surface texture, the key lies in understanding the tactile perception mechanism of the human brain. Human fingertips rely on a large number of tactile sensors to receive surface texture information. In contrast, tactile sensors such as Pacinian Corpuscle are sensitive to vibration signals, which shows that mechanical vibration signals are important for perceiving and distinguishing textures.

## 4 CONCLUSIONS

In this paper, ERPs evoked by the friction withdrawal when exploring the object's surface are studied and analyzed to find out whether the cognition will still occur after the withdrawal of friction. It turns out that P3 of the ERPs were missing when the friction was withdrawn. As a result, it can be considered that the cognitive process doesn’t occur. This paper is of great significance to studying haptic representation and perception of humanoid robots, which contributes to the further study of haptic perception mechanisms in the future.

1. This paper only studies the withdrawal of friction in passive touch. Contrary to passive tactile sense, active tactile sense allows volunteers to produce psychological expectations before exploring the surface characteristics of objects. This distinction has a great influence on some typical components of ERPs and should be discussed.
2. This paper is just an experiment on the dimension of the tactile texture. For volunteers, roughness tests are relatively simple. If you switch to other slightly harder dimensions, it is also worth verifying if the cognitive process continues until the finger leaves the sample.
3. This paper only shows that there is no significant correlation between the withdrawal friction and the cognition through EEG analysis. It is also worth discussing whether the cognitive process of humans will continue to form memories and feedback to P3 for cognition after the withdrawal of friction and complete the complete whole process of cognition.

## CONFLICT OF INTEREST

The authors declare no conflict of interest.

## ETHICS STATEMENT

The study was conducted according to the guidelines of the Declaration of Helsinki and approved by the Institutional Review Board of Xuzhou Central Hospital (protocol code XZXY‐LJ‐20210513‐054 and May 13rd, 2021). Informed consent was obtained from all subjects involved in the study.

## Data Availability

The data that support the findings of this study are available from the corresponding author upon reasonable request
